# Quorum-Sensing Signaling Molecule 2-Aminoacetophenone Mediates the Persistence of Pseudomonas aeruginosa in Macrophages by Interference with Autophagy through Epigenetic Regulation of Lipid Biosynthesis

**DOI:** 10.1128/mbio.00159-23

**Published:** 2023-04-03

**Authors:** Arijit Chakraborty, Asel Kabashi, Samuel Wilk, Laurence G. Rahme

**Affiliations:** a Department of Surgery, Massachusetts General Hospital and Harvard Medical School, Boston, Massachusetts, USA; b Shriners Hospitals for Children Boston, Boston, Massachusetts, USA; c Department of Microbiology, Harvard Medical School, Boston, Massachusetts, USA; National University of Singapore

**Keywords:** *Pseudomonas aeruginosa*, persistence, quorum sensing, 2-aminoacetophenone, macrophages, immunometabolism, histone deacetylation, epigenetic reprogramming, MvfR, PqsR, autophagy, *Scd1*, fatty acids

## Abstract

Macrophages are crucial components of the host’s defense against pathogens. Recent studies indicate that macrophage functions are influenced by lipid metabolism. However, knowledge of how bacterial pathogens exploit macrophage lipid metabolism for their benefit remains rudimentary. We have shown that the Pseudomonas aeruginosa MvfR-regulated quorum-sensing (QS) signaling molecule 2-aminoacetophenone (2-AA) mediates epigenetic and metabolic changes associated with this pathogen’s persistence *in vivo*. We provide evidence that 2-AA counteracts the ability of macrophages to clear the intracellular P. aeruginosa, leading to persistence. The intracellular action of 2-AA in macrophages is linked to reduced autophagic functions and the impaired expression of a central lipogenic gene, stearoyl-CoA desaturase 1 (*Scd1*), which catalyzes the biosynthesis of monounsaturated fatty acids. 2-AA also reduces the expression of the autophagic genes Unc-51-like autophagy activating kinase 1 (*ULK1*) and *Beclin1* and the levels of the autophagosomal membrane protein microtubule-associated protein 1, light chain 3 isoform B (LC3B) and p62. Reduced autophagy is accompanied by the reduced expression of the lipogenic gene *Scd1*, preventing bacterial clearance. Adding the SCD1 substrates palmitoyl-CoA and stearoyl-CoA increases P. aeruginosa clearance by macrophages. The impact of 2-AA on lipogenic gene expression and autophagic machinery is histone deacetylase 1 (HDAC1) mediated, implicating the HDAC1 epigenetic marks at the promoter sites of *Scd1* and *Beclin1* genes. This work provides novel insights into the complex metabolic alterations and epigenetic regulation promoted by QS and uncovers additional 2-AA actions supporting P. aeruginosa sustainment in macrophages. These findings may aid in designing host-directed therapeutics and protective interventions against P. aeruginosa persistence.

## INTRODUCTION

Therapeutic interventions to counter infections caused by ESKAPE pathogens are increasingly diminishing due to the continued rise in antimicrobial resistance (1, 2). Pseudomonas aeruginosa is a recalcitrant ESKAPE pathogen that causes persistent difficult-to-treat community-acquired and nosocomial infections (3–5). Elucidating mechanisms important for the survival and clearance of this pathogen are crucial in developing novel approaches to combat P. aeruginosa infections.

During infection, P. aeruginosa synthesizes and secretes several low molecular weight molecules regulated by cell density-dependent signaling systems, referred to as quorum sensing (QS), to synchronize and effectively modulate bacterial virulence functions (6–9). The P. aeruginosa multiple virulence transcription factor MvfR, also called PqsR, is one of the central QS systems in this pathogen. MvfR directly regulates the transcription of the *pqsABCDE* operon genes (10, 11) that control the synthesis of many small molecules produced and secreted in infected human tissues, including the aromatic volatile compound 2-aminoacetophenone (2-AA) (9, 12). 2-AA is an interkingdom effector molecule that has been shown to promote chronic infection phenotypes via its effects on both the pathogen (13, 14) and the host, leading to the long-term presence of P. aeruginosa in infected tissues (15, 16). Moreover, in previous studies, we have shown the systemic effects of 2-AA in skeletal muscle (17–19). It induces oxidative stress and apoptosis and promotes mitochondrial dysfunction (17, 18, 20). The 2-AA-mediated actions are not only limited to skeletal muscle but are also observed in immune cells. In macrophages, 2-AA dampens cytokine responses through epigenetic reprogramming implicating the histone deacetylase 1 (HDAC1) (21, 22). Epigenetic reprogramming is associated with the long-term presence of P. aeruginosa in infected mice (22).

While P. aeruginosa is considered an extracellular pathogen, an increasing number of studies indicate that this organism can also reside and survive intracellularly, utilizing specific functions (23–25). Macrophages are the first line of defense against pathogens, and they respond efficiently to bacterial invasion by engulfing and killing the bacteria. Many macrophage functions depend on membrane integrity and dynamics. Lipids are shown to be critical in cellular stabilization and signaling (26), and alterations in their composition and distribution can severely affect autophagy (27–29). Substantial contributions to macrophage functions come from the plasma membrane containing many glycoprotein receptors interacting with the membrane’s dynamic microdomains enriched with cholesterol and sphingolipids known as lipid rafts to facilitate cellular signaling (30, 31). These lipid rafts initiate antibacterial responses and are used by intracellular pathogens to enter the cell, propagate, and egress (32). Lipid biosynthesis is essential for membrane remodeling and synthesis of inflammatory mediators in M1 macrophages (33, 34). Although it is clear that macrophages reprogram their lipid metabolism in response to activation signals, knowledge of how different proinflammatory stimuli reshape the macrophage lipidome is limited. It has been shown that Toll-like receptor signals promote divergent fatty acid synthetic programs, including saturated fatty acids (SFAs) and monounsaturated fatty acids (MUFAs) (35). Among these, stearoyl-CoA desaturase 1 (SCD1) is an enzyme responsible for the desaturation of SFA into MUFAs (36).

Studies of P. aeruginosa internalization and clearance have implicated membrane functions (37, 38). While bacterial transport to lysosomal compartments is a well-orchestrated event carried out by various anchor proteins and lipids (39), less is known about the transport of P. aeruginosa and its mechanism of escape from the lysosomal compartments. Indirect evidence points to lipid homeostatic imbalances resulting from P. aeruginosa infection, including the decrease of host unsaturated immunomodulating lipids that result in the sustenance of bacteria inside the host neutrophils (40) and the triggering of the arachidonic acid-dependent inflammatory cascade by ExoU (41). While evidence points toward a role for lipids alterations during P. aeruginosa infection, however, the mechanistic basis of these membrane changes is unclear.

The primary purpose of this study was to interrogate further the 2-AA action on macrophages and their impact on P. aeruginosa persistence. Our findings point to specific actions of this small molecule on these professional phagocytes, involving autophagy and lipid metabolism components. They reveal that the dysregulation of autophagy and lipid metabolism functions implicate chromatin modifications. The results from the present study provide a new understanding of the P. aeruginosa-macrophage interactions and highlight the critical actions of this pathogen’s QS molecule 2-AA in promoting bacterial persistence.

## RESULTS

### 2-AA sustains P. aeruginosa burden in macrophages.

**FIG 1:**
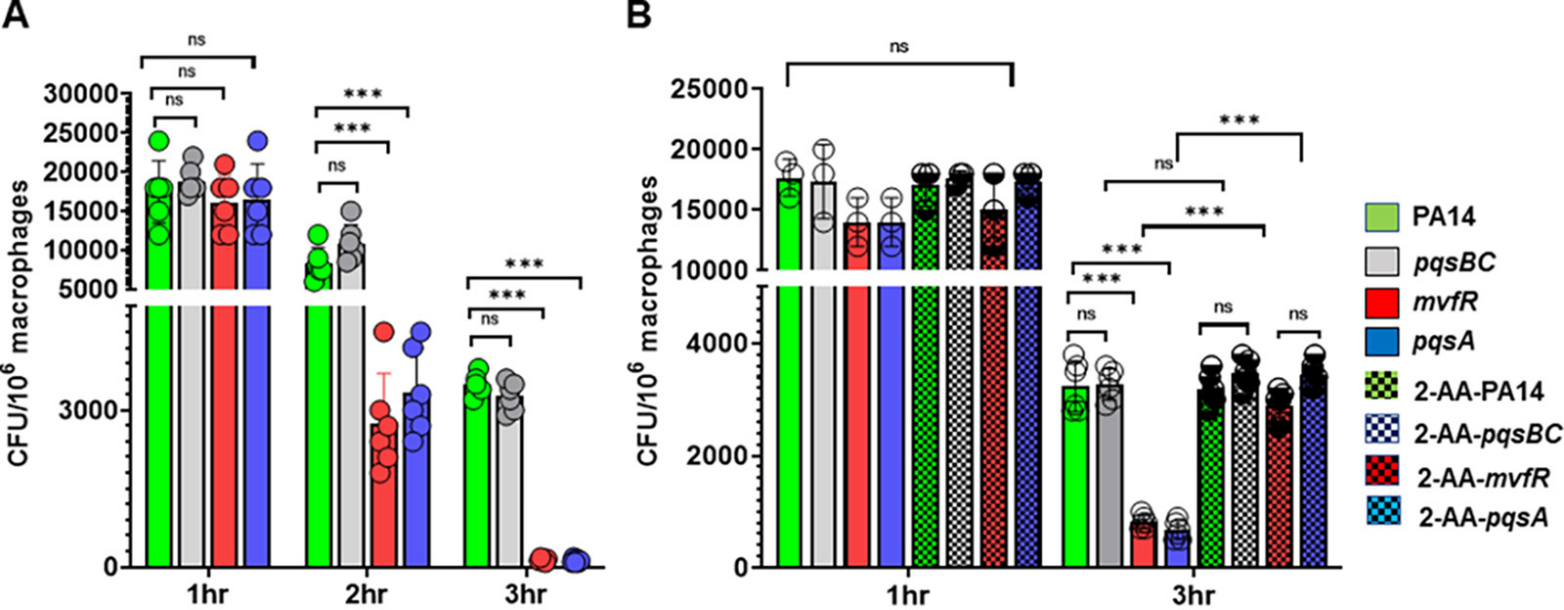
The MvfR-regulated quorum sensing signaling molecule 2-AA sustains P. aeruginosa burden in macrophages. (A) Enumeration of the intracellular P. aeruginosa, as CFU/10^6^ RAW 264.7 macrophages (*mΦ*) at 1, 2, and 3 h postinfection with WT PA14 (green) or the isogenic mutants nonproducing 2-AA *mvfR* (red) and *pqsA* (blue) and the 2-AA producing isogenic mutant *pqsBC* (gray). Bacterial loads were significantly reduced in *mvfR* and *pqsA* mutants relative to PA14 and *pqsBC*. (B) Enumeration of the P. aeruginosa intracellular CFU/10^6^ RAW 264.7 *mΦ* in the absence or presence of 200 μM 2-AA (dotted) at 1 h and 3 h postinfection with PA14 or mutants *mvfR*, *pqsA*, or *pqsBC*. The addition of 2-AA significantly increases the intracellular bacterial load in *mvfR* and *pqsA*-infected macrophages relative to those infected with these nonproducing 2-AA mutants at 3 h postinfection. The error bars denote ± SD. One-way ANOVA followed by Tukey posttest was applied. ***, *P* < 0.001; ns indicates no significant difference. Data represent *n* ≥ 3 independent replicates. Each circle represents data from one replicate.

The P. aeruginosa MvfR-regulated QS signaling molecule 2-AA contributes to the persistence of this pathogen in host tissues (20, 21). Since macrophages are the main phagocytes of the innate immune system and are considered key players in bacterial clearance, we sought to determine the role of these immune cells in permitting the 2-AA-promoted persistence in infected host tissues. Using the P. aeruginosa clinical isolate PA14 (wild type [WT]) and the isogenic QS mutants *mvfR* and *pqsA* that do not produce 2-AA and mutant *pqsBC* that produces 2-AA but not hydroxyquinolines (14), we infected murine bone marrow-derived macrophages (BMDM). Similarly to *mvfR* and *pqsA* mutants, the *pqsBC* mutant does not produce hydroxyquinolines (14). We performed gentamicin protection assay studies to determine the intracellular presence of these bacterial strains over the course of 3 h. We found that the intracellular burden in the BMDM cells infected with *mvfR* and *pqsA* mutants significantly decreased by 2 h and almost cleared by 3 h, relative to cells infected with PA14 (Fig. 1A). In contrast, macrophages infected with PA14 or isogenic *pqsBC* mutant sustained similar and significant bacterial burdens throughout the course of infection. Exogenous addition of 2-AA (200 μM) to RAW264.7 cells infected with the 2-AA-deficient strains *mvfR* or *pqsA* restored the intracellular bacterial burden to levels similar to those observed in cells infected with PA14 and *pqsBC* (Fig. 1B). The supplementation of 2-AA in PA14- or *pqsBC*-infected macrophages did not have any additive effect on the intracellular bacterial load. These findings strongly support 2-AA’s role in interfering with the ability of these professional phagocytes to clear the presence of P. aeruginosa.

### Impairment of autophagy contributes to the P. aeruginosa intracellular bacterial load in infected macrophages.

**FIG 2:**
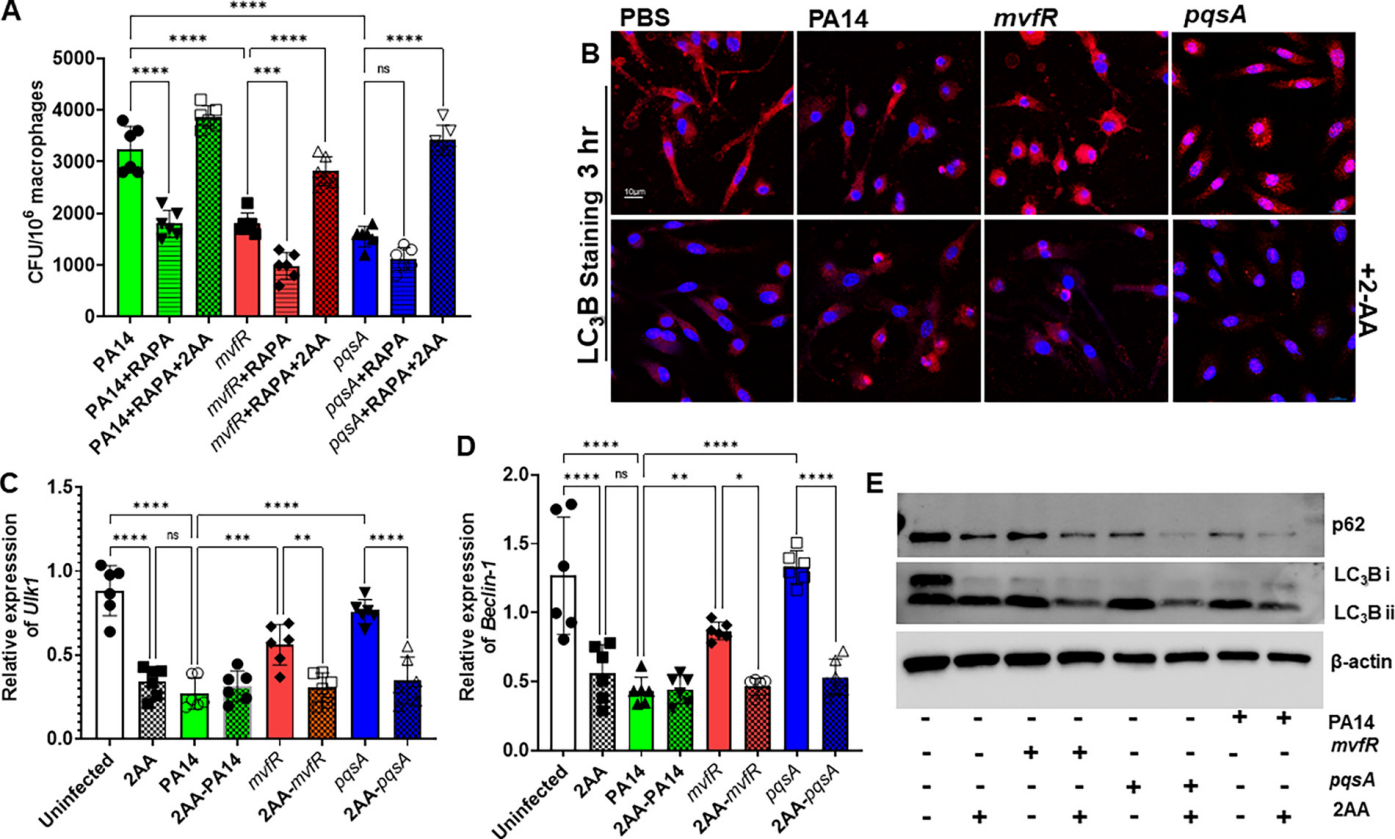
Impairment of autophagy contributes to the P. aeruginosa intracellular bacterial load in infected macrophages. (A) Enumeration of the intracellular P. aeruginosa CFU/10^6^ RAW264.7 *mΦ* at 3 h postinfection with PA14 (green) strain or the isogenic mutants nonproducing 2-AA *mvfR* (red) and *pqsA* (blue) in the absence or in the presence of exogenously added 200 μM 2-AA (dotted), or 10 mM rapamycin (striped). The addition of the autophagic inducer rapamycin significantly decreases the intracellular CFU in PA14-infected macrophages (green striped) and isogenic mutants *mvfR* (red striped) compared to infected macrophages that did not receive rapamycin (solid color). This decrease is abolished in all macrophage-infected groups treated with 2-AA (dotted colors), despite the presence of rapamycin (striped colors). (B) Representative confocal images depict LC_3_B puncta (red) in BMDM cells infected with PA14, *mvfR*, or *pqsA* (top). PBS was used as a control. PA14-infected macrophages show fewer LC_3_B puncta than macrophages infected with *mvfR* and *pqsA*. However, supplementation with 2-AA (200 μM) in macrophages infected with *mvfR* and *pqsA* (bottom) also showed lower LC_3_B punctation than their nonsupplemented counterparts. The experiment was repeated independently three times with similar results. (C and D) RT-qPCR analysis of PA14 (green)-infected RAW264.7 macrophages reveal significantly lower *ULK1* and *Beclin1* expression levels relative to *mvfR* (red)- or *pqsA* (blue)-infected macrophages, while 2-AA (200 μM) addition in both *mvfR* (red dotted)- and *pqsA* (blue dotted)-infected cells decreases the expression of these genes. The error bars denote ± SD. One-way ANOVA followed by Tukey posttest was applied. *, *P* < 0.05; **, *P* < 0.01; ***, *P* < 0.001; ****, *P* < 0.0001; ns indicates no significant difference. Data represent *n* = 6 independent replicates. Each dot represents data from one replicate. (E) Representative immunoblot of p62, LC_3_B protein levels in PA14-, *mvfR*-, or *pqsA*-infected macrophages ± 2-AA (200 μM). β-Actin was used as a control. The blots are representative of three independent experiments.

The 2-AA-mediated sustainment of the intracellular bacterial load prompted us to investigate whether 2-AA impacts autophagy. First, we used the autophagy inducer rapamycin to examine the contribution of autophagy in the 2-AA-mediated reduced intracellular bacterial clearance. Pretreatment of RAW264.7 cells with rapamycin starting 3 h before infection significantly decreased the intracellular bacterial load in cells infected with the strain PA14 and the isogenic mutants lacking 2-AA production (Fig. 2A). The exogenous addition of 2-AA to these cells increased the bacterial intercellular load, annulling the observed rapamycin effect. Subsequently, we performed punctate and diffuse staining of BMDM cells using the key autophagosomal membrane protein microtubule-associated protein 1, light chain 3 isoform B (LC_3_B) (Fig. 2B). Fluorescence confocal microscopy images of the infected BMDM (Fig. 2B) and quantification (Fig. S1 in the supplemental material) showed significantly lower (~3-fold) LC3B punctation in PA14 versus *mvfR* or *pqsA* infected cells. However, the exogenous addition of 2-AA in BMDM cells infected with *mvfR* or *pqsA* reduced the LC3B punctation to the levels observed in the cells infected with PA14 (Fig. 2B, bottom; Fig. S1). Consistent with these findings, the expression of the autophagic genes *ULK1* (Unc-like kinase-1) and *Beclin1* were significantly lower (~2.8-fold for *ULK1* and ~2.7-fold for beclin1) in macrophages infected with PA14 compared to the levels observed in uninfected cells or those infected with mutants *pqsA* or *mvfR*. In corroboration, the expression of *ULK1* and *Beclin1* decreased following the addition of 2-AA in *pqsA*- or *mvfR*-infected macrophages, mimicking those observed in cells infected with PA14 (Fig. 2C and D). Similarly, Western Blot analyses of the LC3B autophagic protein and autophagy-specific substrate p62 from whole-cell lysates 3 h postinfection showed that in the presence of 2-AA, the proteins levels of LC3BII and p62 were hampered, further supporting that this small molecule promotes autophagic dysfunction (Fig. 2E).

10.1128/mbio.00159-23.1 FIG S1 Graphical intensity plots of Fig. 2B showing arbitrary fluorescent units depicting the intensity of LC_3_B versus the intensity of DAPI in 2-AA treated or infected BMDM cells. A significant reduction in LC_3_B staining relative to the PBS-treated cells is observed within 3 h post-2-AA treatment. PA14-infected macrophages (green) have less LC_3_B staining than macrophages infected with *mvfR* (red) or *pqsA* (blue). However, supplementation with 2-AA (200 μM) in cells infected with *mvfR* and *pqsA* (checkered pattern) showed lower LC3B punctation than their nonsupplemented counterparts. Data represent *n* = 3 independent replicates. Each circle represents data from at least 2 replicates. The error bars denote ± SD. One-way ANOVA followed by Tukey posttest was applied. **, *P* < 0.01; ***, *P* < 0.001. Download FIG S1, TIF file, 0.1 MB. Copyright © 2023 Chakraborty et al. 2023 Chakraborty et al. https://creativecommons.org/licenses/by/4.0/ This content is distributed under the terms of the Creative Commons Attribution 4.0 International license.

### 2-AA impacts membrane lipids and decreases lipogenic gene expression.

**FIG 3:**
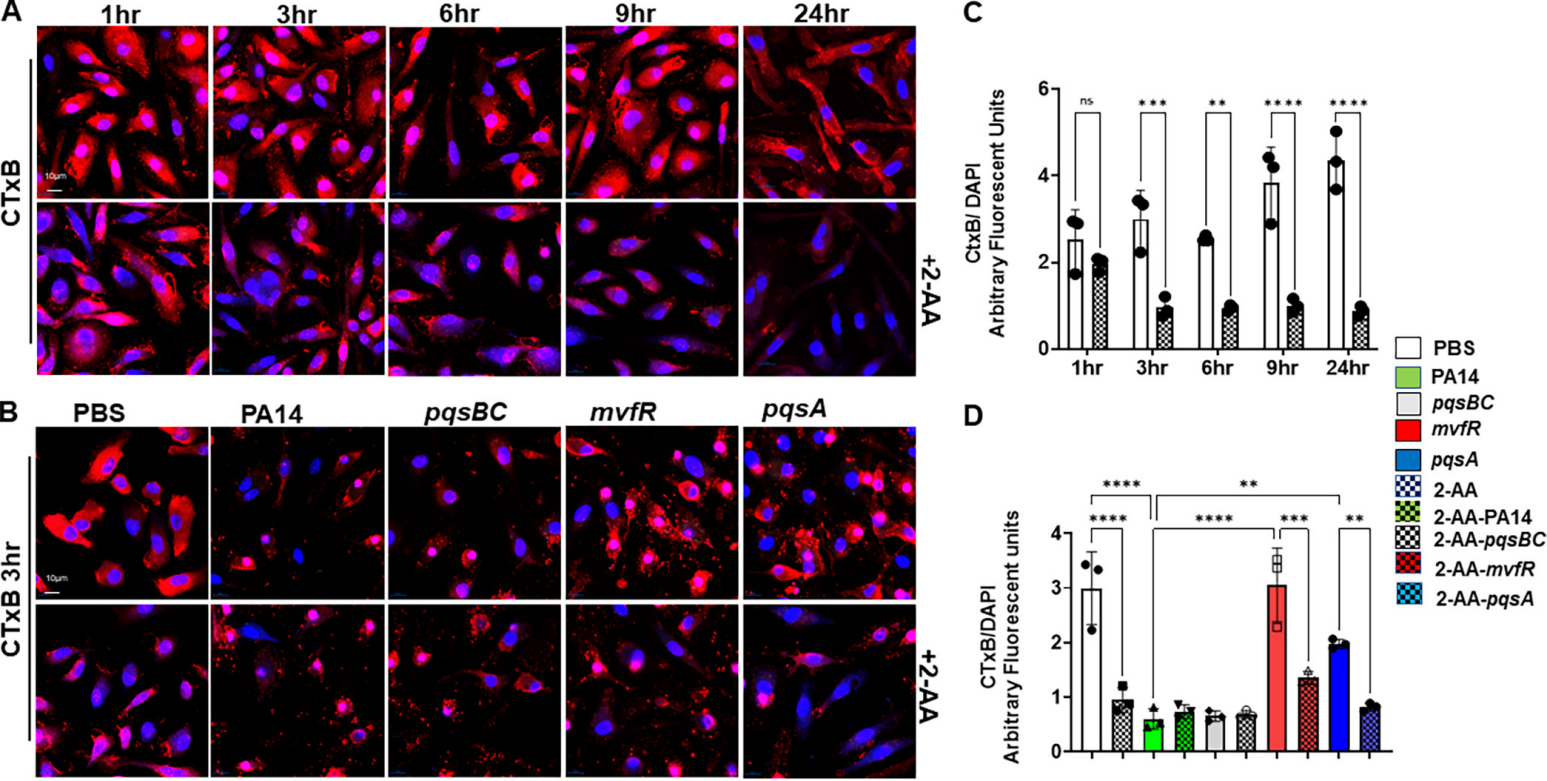
2-AA affects membrane lipids. Representative confocal images of BMDM cells depicting membrane ganglioside staining with fluorescent-labeled cholera toxin B (red) (A) untreated (top) or treated (bottom) with 2-AA (200 μM) for 1 h, 3 h, 6 h, 9 h, and 24 h and infected (B) with PA14, *mvfR*, *pqsA*, and *pqsBC* (top), or treated with 2-AA (bottom) for 3 h. (C and D) Corresponding graphical intensity plots of panels A and B, respectively. Arbitrary fluorescent units depict the intensity of membrane staining (red) versus the intensity of DAPI (blue). Significant reduction in membrane staining relative to the PBS-treated cells is observed within 3 h post-2-AA treatment. The error bars denote ± SD. One-way ANOVA followed by Tukey posttest was applied. *, *P* < 0.05; **, *P* < 0.01; ***, *P* < 0.001; ****, *P* < 0.0001; ns indicates no significant difference. The images are representative of three independent experiments.

Autophagy is an active cellular process requiring interaction between membrane lipids and the autophagic machinery proteins (42, 43). Therefore, we assessed the effect of 2-AA on the BMDM membrane using fluorescent-labeled cholera toxin B (CTx-B) (31), which binds tightly to its receptor, the GM1 glycosphingolipids and is often used as a probe of membrane biology and marker of lipid rafts (43, 44). Since membrane alteration may be a dynamic event, we performed confocal microscopy to evaluate the potential effect of 2-AA on the membrane of fixed BMDM cells at different time points (3 h, 6 h, 9 h, and 24 h). The fluorescence confocal microscopy images (Fig. 3A) and quantification (Fig. 3B) show that the infected BMDM cells receiving 2-AA exhibit a significant reduction (~3-fold) in membrane staining starting as early as 3 h post-2-AA addition compared to the uninfected cells. A similar significant reduction (~5-fold) in staining was observed in cells infected with the 2-AA-producing strain PA14 and mutant strain *pqsBC* but appeared not as prominent with the non-2-AA-producing mutant strains *mvfR* and *pqsA* (Fig. 3C and D). RAW 264.7 macrophages infected with PA14 or the *mvfR* with or without exogenous addition of 2-AA gave similar CTx-B staining results to BMDM cells (Fig. S2C).

**FIG 4:**
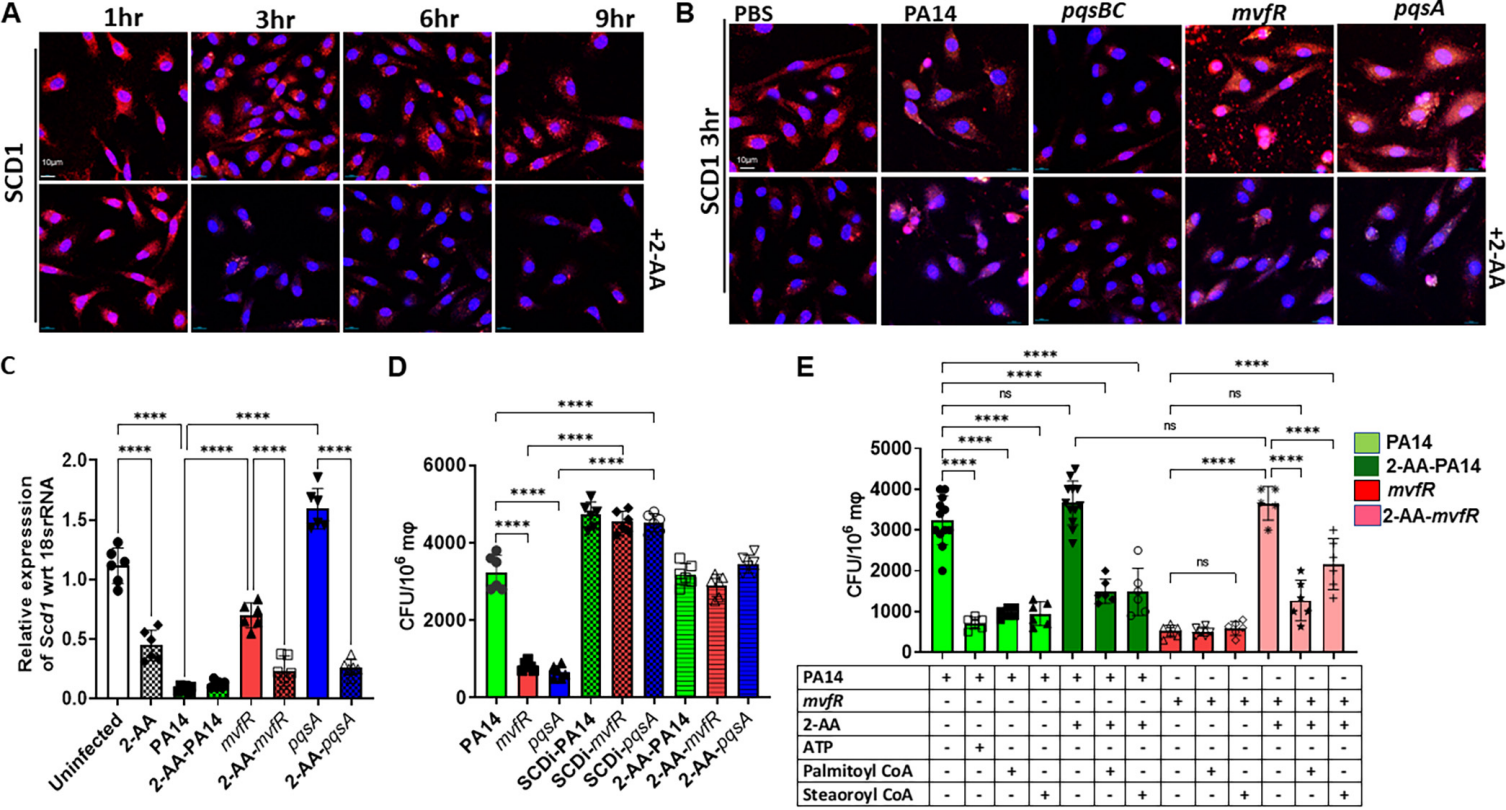
2-AA affects lipogenic gene expression in macrophages. (A) Representative confocal images of SCD1 immunocytochemical staining (red) in BMDM treated (bottom)/untreated (top) with 2-AA. The macrophages were counterstained with DAPI (Blue). (B) Representative confocal images showing SCD1 expression (red) in BMDM infected with PA14, *mvfR*, *pqsA*, and *pqsBC* (top). Supplementation of 2-AA (200 μM) in macrophages infected with *mvfR* or *pqsA* (bottom) showed lower SCD1 staining than their nonsupplemented counterparts. (C) RT-qPCR analysis of *Scd1* gene expression in RAW264.7 *mΦ* infected with PA14 (green), *mvfR* (red), or *pqsA* (blue). Supplementation of 2-AA in infected cells with *mvfR* (red dotted), *pqsA* (blue dotted), or uninfected (white dotted) cells decreases *Scd1* expression levels. (D) Bacterial load represented as CFU/10^6^ mΦ in the absence and presence of 10 μM SCD1 inhibitor (SCD1i) or 200 μM 2-AA. The bacterial load increases when infected RAW 264.7 cells are treated with either SCD1i or 2-AA. (E) Enumeration of PA14 and *mvfR* intracellular CFU/10^6^ RAW 264.7 mΦ cells in the absence and presence of palmitoyl-CoA (50 μM) and stearoyl-CoA (50 μM). The addition of these compounds decreases the intracellular bacterial load in the presence of 2-AA. Addition of ATP (10 μM) was used as a control in PA14 cells. Data represent *n* ≥ 5 independent replicates. Each point represents data from one replicate. The error bars denote ± SD. One-way ANOVA followed by Tukey’s honestly significant difference (HSD) *post hoc* test was applied; ****, *P* < 0.0001; ns indicates no significant difference.

10.1128/mbio.00159-23.2 FIG S2 Representative graphical intensity plots corresponding to Fig. 4A, respectively, depicting intensity over time of SCD1 (red) versus intensity of DAPI (blue) in BMDM cells treated (A) with 2-AA (white checkered/untreated white) and infected (B) with PA14 (green), *mvfR* (red), *pqsA* (blue), and *pqsBC* (gray) or infected with these strains and supplemented with 2-AA (200 μM) (checkered). Data represent *n* = 3 independent replicates. Each dot represents data from at least 2 replicates. The error bars denote ± SD. One-way ANOVA followed by Tukey’s HSD *post hoc* test was applied. **, *P* < 0.01; ns indicates no significant difference. Representative confocal microscopy images depicting staining with CTxB (red) (C) and SCD1 (red) (D) in RAW264.7 cells uninfected (control) and infected with PA14 or *mvfR* in the absence (top) and the presence of exogenously added 2-AA (400 μM) (bottom). Macrophage nuclei were counterstained with DAPI (blue). The images are representative of three independent experiments. Download FIG S2, TIF file, 0.6 MB. Copyright © 2023 Chakraborty et al. 2023 Chakraborty et al. https://creativecommons.org/licenses/by/4.0/ This content is distributed under the terms of the Creative Commons Attribution 4.0 International license.

Membrane alterations in response to various stimuli depend heavily on the structural and functional aspects of their lipids and homeostatic levels of unsaturated fatty acids (45). Therefore, the observed alteration in the BMDM cell membranes suggested that fatty acid metabolism is likely affected (46). Accordingly, we interrogated the involvement of SCD1, a key enzyme in fatty acid metabolism catalyzing the biosynthesis of MUFAs also shown to be constituents of the cell membrane (47). SCD1 uses the preferred substrates palmitoyl coenzyme A (palmitoyl-CoA) and stearoyl coenzyme A (stearoyl-CoA) to form the MUFAs, palmitoleoyl-CoA and oleyl-CoA, two major constituents of membrane phospholipids. BMDM cells treated with 2-AA or infected with the 2-AA-producing strains PA14 or mutant *pqsBC* showed a significant reduction (~4-fold) in SCD1 staining starting at 3 h compared to the cells not receiving 2-AA (Fig. 4A; Fig. S2A) or infected with the mutant strains *mvfR* or *pqsA* (Fig. 4B; Fig. S2B). Further confirmation of these findings is provided by the exogenous addition of 2-AA in *mvfR*- or *pqsA*-infected macrophages where SCD1 staining is almost abolished (Fig. 4B, bottom). Similar results were obtained using RAW 264.7 macrophages (Fig. S2D). Consistent with these findings, *Scd1* transcript levels in RAW 264.7 macrophages were significantly reduced in the presence of 2-AA (Fig. 4C). Notably, such reduction was accompanied by increased P. aeruginosa intracellular CFU in macrophages (Fig. 4D). Using an SCD1 inhibitor (ab142089) reverted the increased bacterial intracellular burden observed with non-2-AA-producing strains *mvfR* and *pqsA*, further strengthening the above findings and implicating the lipogenic enzyme SCD1 in the 2-AA-impeded bacterial clearance.

Given that derangement of *Scd1* gene expression is accompanied by increased levels of intracellular bacterial burden in macrophages and that *Scd1* utilizes the coenzymes palmitoyl-CoA and stearoyl-CoA to catalyze the biosynthesis of MUFAs (46), we hypothesized that addition of these substrates might impede the 2-AA effect in increasing the intracellular bacterial burden. We added 50 μM either stearoyl-CoA or palmitoyl-CoA 3 h prior to infection with PA14 or *mvfR* of RAW 264.7 macrophages with and without the addition of exogenous 2-AA at the time of infection and assessed the bacterial intracellular load (Fig. 4E). ATP, which has been known to induce bacterial killing in macrophages, was used as a control of the PA14 infected cells. Indeed, the PA14-infected macrophages that received stearoyl-CoA, palmitoyl-CoA, and ATP had a significantly lower number of PA14 compared to the infected-only macrophages even when 2-AA is added exogenously in these groups (Fig. 4E). More importantly, however, the addition of stearoyl-CoA, palmitoyl-CoA in *mvfR*-infected macrophages that received 2-AA exogenously decreased significantly the number of intracellular bacteria compared to the *mvfR*-infected macrophages in the presence of 2-AA (Fig. 4E). These results reaffirm the importance of these lipids in the reduction of the 2-AA-mediated intracellular P. aeruginosa burden in macrophages.

### Impairment of lipid homeostasis and autophagy by 2-AA is HDAC1 dependent.

**FIG 5:**
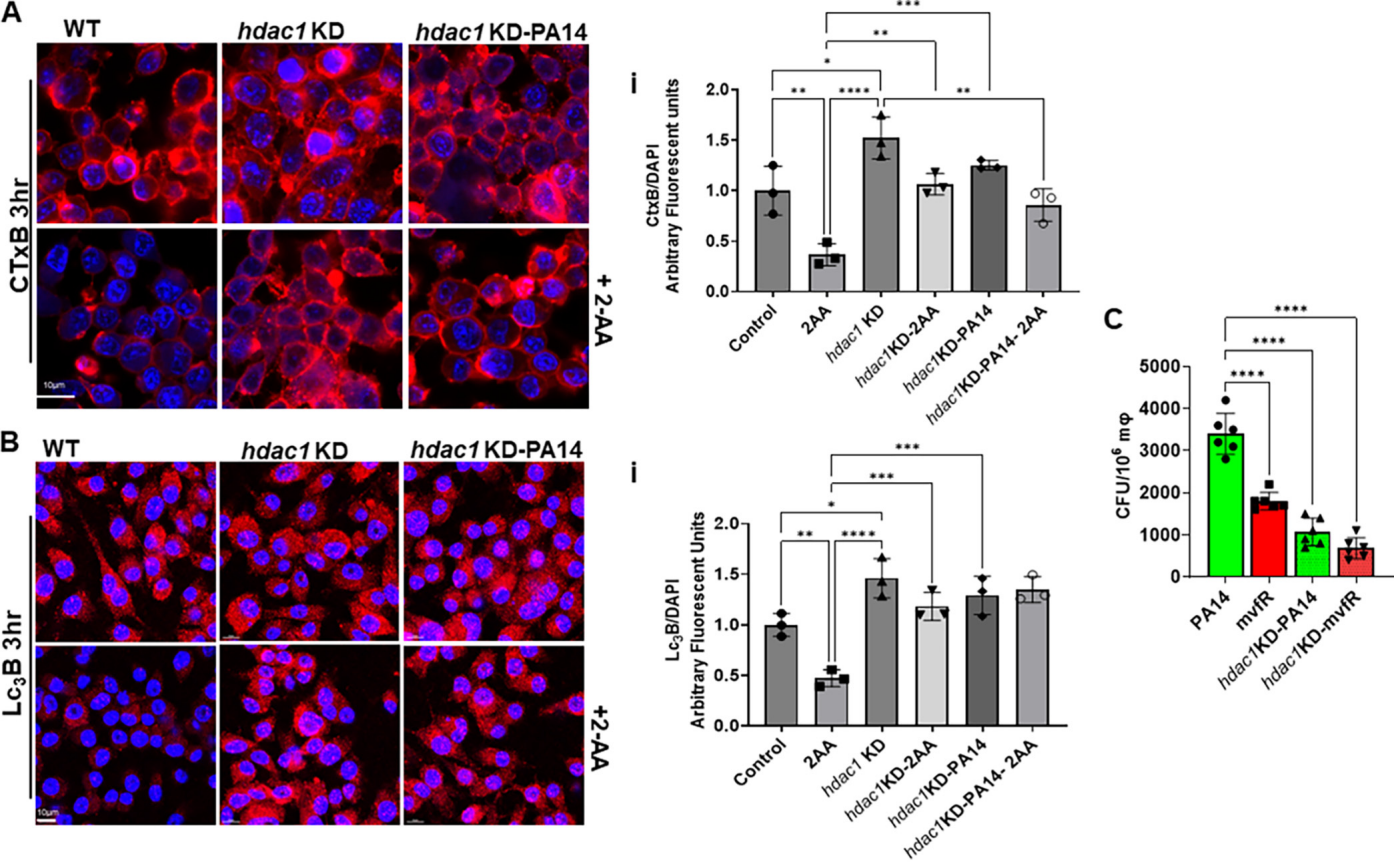
Impairment of lipid homeostasis and autophagy by 2-AA is HDAC1-dependent. (A and B) Representative confocal images and graphical intensity plots (Ai and Bi) depicting enhanced membrane lipid staining with fluorescent-labeled cholera toxin B (red) (A) or LC_3_B puncta (Red) (B) in RAW 264.7 and *hdac1* KD isogenic cells uninfected and infected with PA14 (top) and/or treated with 2-AA (bottom). (C) Enumeration of PA14 intracellular CFU/10^6^ in RAW 264.7 or *hdac1* KD *mΦ* cells. Loss of HDAC1 clears PA14 more efficiently than the wild-type macrophages. Data represent *n* = 6 independent replicates. Each dot represents data from one replicate. The error bars denote ± SD. One-way ANOVA followed by Tukey posttest was applied. *, *P* < 0.05; **, *P* < 0.01; ***, *P* < 0.001; ****, *P* < 0.0001; and ns indicates no significant difference.

Given that 2-AA-mediated immune modulation and associated P. aeruginosa persistence in infected tissues implicates histone acetylation via HDAC1 (20), we examined the relevance of HDAC1 in the observed membrane effects observed, autophagy, and bacterial clearance. To verify whether 2-AA influences these processes in an HDAC1-dependent manner, we first compared CTx-B-stained membranes of RAW264.7 and isogenic *hdac1*-knockdown (KD) cells infected with PA14 or following 2-AA addition. Compared to the control cells receiving 2-AA, HDAC1 KD cells showed robust CTx-B staining, which was not affected by 2-AA addition or PA14 infection (Fig. 5Ai). Similarly, fluorescence confocal microscopy (Fig. 5B) and quantification (Fig. 5Bi) of the LC_3_B punctation showed that punctation was not altered in HDAC1 KD cells following infection or 2-AA exogenous addition compared to wild-type cells (2-fold reduction in LC_3_B). The effects observed in HDAC1 KD infected cells were accompanied by a significantly reduced (~3-fold decrease) bacterial load than that found in control isogenic RAW264.7-infected macrophages (Fig. 5C), indicating that 2-AA alters the clearance potential of macrophages in an HDAC1-dependent manner.

### HDAC1-mediated deacetylation of H3K18 at the promoter sites of *Beclin1* and *Scd1* genes decreases their expression.

**FIG 6:**
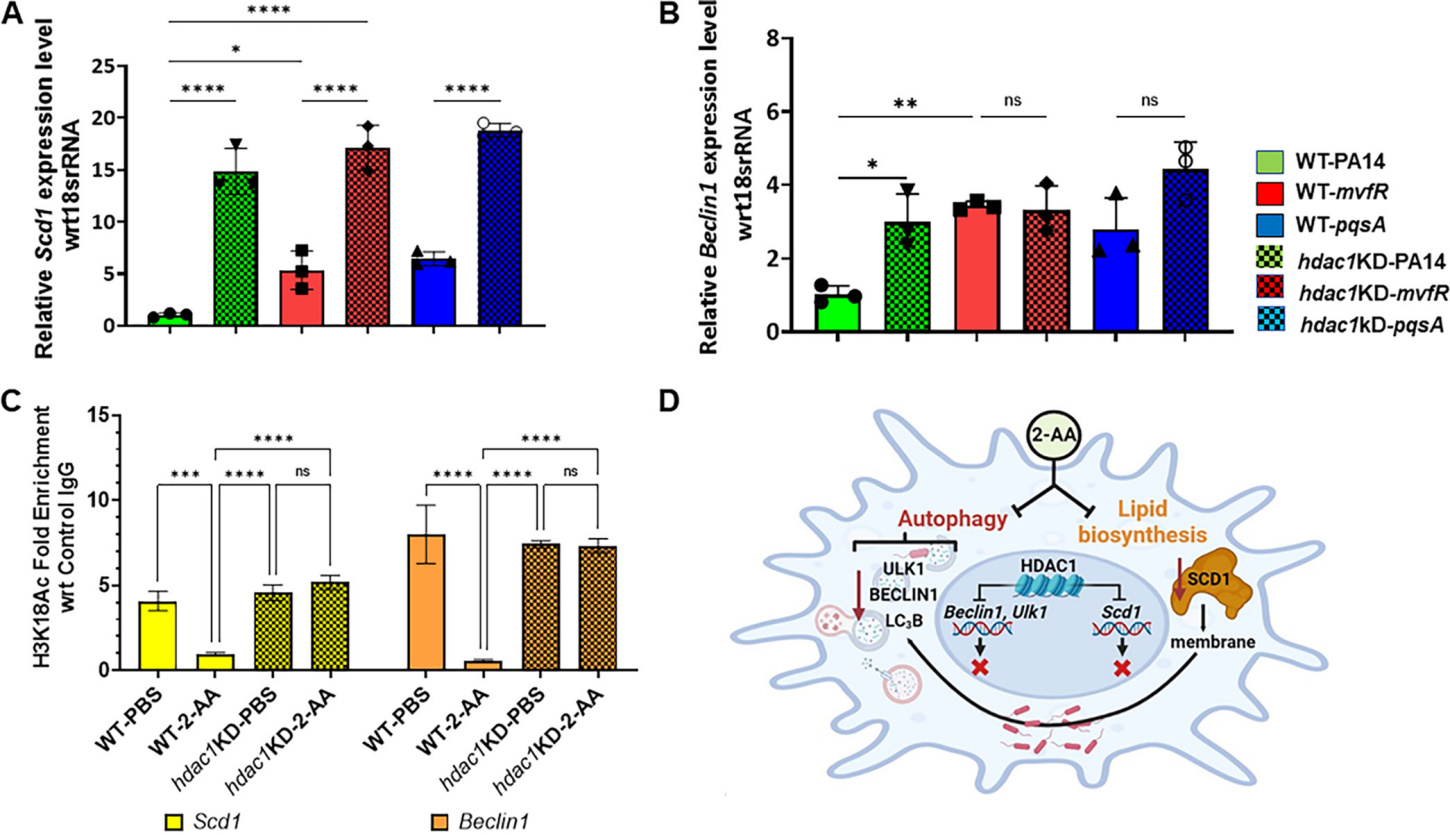
HDAC1-mediated deacetylation of H3K18 at the promoter of *Beclin1* and *Scd1* decreases their gene expression. Analysis of *Scd1* (A) and *Beclin*1 (B) transcript levels by RT-qPCR. Shown is the relative gene expression in PA14 (green)-, *mvfR* (red)-, or *pqsA* (blue)-infected RAW 264.7 and *hdac1* KD cells (dotted bars) compared to uninfected cells. The gene expression levels of *Scd1* and *Beclin1* in *hdac1* KD cells (dotted) are significantly higher than in RAW 264.7-infected cells (plain). (C) Occurrence of H3K18ac marks on the Beclin1 and Scd1 promoters. ChIP-qPCR was used to show the enrichment of H3K18ac marks on the *Beclin1* (orange) promoter and *Scd1* promoter (yellow) in RAW 264.7 cells treated with 2-AA (400 mM) compared to untreated macrophages or *HDAC1*-knockdown treated macrophages. Each circle represents data from one replicate. The error bars denote ± SD. One-way ANOVA followed by Tukey’s HSD posttest was applied. *, *P* < 0.05; **, *P* < 0.01; ***, *P* < 0.001; ****, *P* < 0.0001; ns indicates no significant difference. (D) Schematic representation of 2-AA effects on macrophages. Once P. aeruginosa is present in macrophages, 2-AA secures its intracellular sustenance through changes in the host autophagy by affecting autophagic nucleation and lipid biosynthesis. Alteration in macrophage lipid homeostasis results from changing the expression of the key lipogenic gene *Scd1*, which also affects the macrophage membrane, possibly through reduced monounsaturated fatty acid (MUFA) biosynthesis. The epigenetic eraser HDAC1 mediates inhibition of MUFAs biosynthesis and autophagy.

Low intracellular bacterial load, robust LC_3_B punctation, and CTx-B staining in RAW 264.7 isogenic *HDAC1* KD-infected cells prompted us to assess the expression of *Scd1* and *Beclin1* genes, whose products are known to play central roles in autophagy. As shown in Fig. 6A, following infection with PA14, *mvfR*, or *pqsA*, the transcript levels of *Scd1* were significantly higher in *HDAC1* KD cells than in corresponding infected control macrophage cells. Moreover, the expression levels of *Beclin1* in *HDAC1* KD-infected cells with all strains used were higher than in control cells infected with the PA14 strain (Fig. 6B). To determine whether 2-AA alters gene expression by impacting histone marks at the promoters of *Scd1* and *Beclin1*, we performed chromatin immunoprecipitation-quantitative PCR (ChIP-qPCR). Enrichment of H3K18ac, specifically at the promoter region of *Scd1* and *Beclin1*, was significantly attenuated in 2-AA treated control cells compared to the untreated control cells (Fig. 6C). In contrast, in *HDAC1* KD cells, similar enrichment of H3K18 acetylation at the promoter region of both *Scd1* and *Beclin1* was observed regardless of the 2-AA addition.

## DISCUSSION

This study provides novel insights into the actions of P. aeruginosa in offsetting its clearance by macrophages. We show that the MvfR-regulated QS signaling molecule 2-AA impacts the autophagic machinery and lipids biosynthesis to sustain the presence of P. aeruginosa in macrophages after being engulfed by phagocytosis. A series of infection studies in BMDM and RAW 264.7 cells using the WT P. aeruginosa clinical isolate PA14 and isogenic mutants indicate that 2-AA is a contributing culprit of these dysfunctions. A 2-AA-mediated autophagic dysfunction is supported by the decreased expression of the autophagic genes *ULK1* and *Beclin1*, along with the reduced levels of the LC_3_B protein and the autophagy-specific substrate p62. Our results show that in the presence of 2-AA, the rapamycin-induced autophagy effect is annulled. Rapamycin is an inducer of autophagy and an inhibitor of the mechanistic target of rapamycin complex 1 (mTORC1). Inhibition of mTORC1 is required to initiate the autophagy process. (48) P. aeruginosa has been reported to survive in epithelial cells by the ExoS-mediated simultaneous inhibition of both the mTOR pathway and autophagy through independent mechanisms (49) 2-AA’s ability to interfere in autophagy may involve mTOR signaling. Future studies aimed at exploring this aspect further are expected to be informative. Nevertheless, adding rapamycin further strengthens the 2-AA’s negative effect on autophagy initiation. At the same time, using the 2-AA-deficient mutant *mvfR* in this setting also suggests that additional 2-AA independent mechanism(s) impact the autophagic machinery.

The reduced expression of the lipogenic gene SCD1 and the membrane perturbations seen through the reduced SCD1 and CTx-B membrane staining of the BMDM cells infected with the PA14 strain pointed to dysfunctional fatty acid metabolism. The degree of unsaturation in membrane lipids is controlled partly by SCD1. Inhibition of SCD1 function in macrophages infected with PA14 isogenic non-2-AA-producing mutants increases the intracellular bacterial burden. In contrast, adding the SCD1 substrates palmitoyl-CoA and stearoyl-CoA reduces the bacterial burden in these macrophages, providing strong evidence of 2-AA’s impact on lipid biosynthesis. The 2-AA-dependent decrease of CTx-B in infected macrophages signifies the reduced ganglioside GM1 in the plasma membrane. However, since CTx-B binds to the sugar component of gangliosides, glycosylation may also be suppressed by 2-AA. Future studies are planned to address this aspect. Overall, the findings presented here offer novel insights into the actions of P. aeruginosa and provide strong evidence of 2-AA’s impact on fatty acid metabolism and its importance in the clearance of this pathogen.

The importance of fatty acids in P. aeruginosa infection has been previously reported. For example, oleate was shown to protect Caenorhabditis elegans from this pathogen’s infection (50). In addition, P. aeruginosa enzymes, including phospholipase C, lipoxygenase A, and ExoU, have been reported to impact host lipid pathways in the lung epithelial cells (51). The P. aeruginosa QS autoinducer N-3-oxo-dodecanoyl homoserine lactone has been reported to change the membrane dipole potential in lymphocytes by accumulating in the cholesterol-containing microdomains, leading to membrane conformation changes that impact immune signaling (52). Macrophage functions affecting P. aeruginosa clearance have also been reported. Loss of the protease-activated receptor 2 (PAR2) impairs the clearance of this pathogen in alveolar macrophages. PAR2 promotes P. aeruginosa clearance by inducing cAMP levels, showing that the cAMP-Rac1 signaling cascade activates the phagocytosis of this pathogen in alveolar macrophages (53). The nuclear hormone receptor peroxisome proliferator-activated receptor gamma (PPARg) also comes into play in P. aeruginosa clearance in mice lungs as it induces paraoxonase, an enzyme involved in hydrolysis and degradation of the QS homoserine lactones (54). Our results add to the limited repertoire of P. aeruginosa virulence functions and actions identified to counteract its clearance.

Another key finding in our study is the HDAC1 dependency of 2-AA-mediated effect on membrane lipids and autophagy. We show that 2-AA alters the killing potential of macrophages in an HDAC1-dependent manner by demonstrating that *HDAC1* KD restores P. aeruginosa clearance in macrophages. HDACs catalyze the removal of acetyl groups from lysines on histones, increasing chromosome condensation and inhibiting transcription. Indeed, we find HDAC1 enrichment at *Beclin1* and *Scd1* promoters in support of the observed decreased expression of these genes. Our results are consistent with our previous findings on the HDAC1-mediated reprogramming of immune cells found to be associated with the sustained bacterial burden in host tissues (20, 22). The present findings provide new insights into 2-AA-mediated epigenetic reprogramming outcomes and reveal mechanistic actions and players contributing to P. aeruginosa persistence. The reported lipid and autophagic reprogramming in macrophages by a bacterial QS signaling molecule opens new avenues for designing and developing new therapeutics and interventions that may protect patients from recalcitrant persistent infections.

## MATERIALS AND METHODS

### Generation of bone marrow-derived macrophages.

BMDMs were generated by collecting femur and tibia from the hind legs of 6- to 8-week-old CD1 male mice (Charles River). Muscles attached to bones were removed using sterile scissors and forceps. Bone marrow was isolated by flushing the marrow into a sterile 50-mL tube with a syringe filled with RPMI 1640 medium. Upon centrifugation at 2,000 rpm for 5 min, cells were resuspended in RPMI 1640 medium supplemented with 10% FBS, macrophage colony-stimulating factor (M-CSF; 20 ng/mL; StemCell Technologies), 2% penicillin/streptomycin, and 2 mM l-glutamine (Gibco). Subsequently, the cells were plated on 90-mm nontissue culture-treated plates at a density of 5 × 10^6^ cells/mL and incubated at 37°C in a 5% CO_2_ atmosphere. Three days after cell seeding, an extra 10 mL of fresh RPMI 1640 (supplemented with 10% FBS, M-CSF [20 ng/mL], 2% penicillin/streptomycin, and 2 mM l-glutamine) was added to each plate, and incubation continued for an additional 3 days. On the sixth day, supernatants were discarded. The attached cells were resuspended in RPMI 1640 after being dislodged by trypsin-EDTA. Finally, the cells were counted, and approximately 1.5 × 10^6^ cells were seeded in tissue culture plates for 24 h before the experimental procedure. The culture medium was replaced by RPMI 1640 growth medium with 10% FBS.

### Cell cultures.

The mouse macrophages cell line RAW264.7 (ATCC) was grown in DMEM medium, supplemented with 10% heat-inactivated FBS (endotoxin-free Certified FBS; Invitrogen), 2% penicillin/streptomycin, 2 mM l-glutamine, and 10 mM HEPES (all from Gibco). The cells were seeded in T-75 tissue culture flasks (Falcon, USA) and used between passages 2 and 3. For quality control, we tested the cells for mycoplasma with the PlasmoTest kit (Invivogen). *HDAC1* knockdown cells were generated by shRNA and validated by Western blotting analyses as described in our previous publication (20) and grown in a complete DMEM medium in the presence of puromycin (10 μg/mL). The primary antibody to detect HDAC1 was specific to HDAC1 (cat no. 5356; Cell Signaling Technology).

### P. aeruginosa strains and cell infection.

The P. aeruginosa clinical isolate PA14 (Rahme Lab) (55) and isogenic mutants *mvfR*, *pqsA*, *or pqsBC* were used (Rahme lab) (10, 14, 56, 57). The bacteria were grown at 37°C in Luria-Bertani (LB) broth under shaking and aeration overnight, diluted to 1:50,000,000 in fresh LB medium, and grown to an optical density at 600 nm (OD_600nm_) of 2.0. Bacterial cells were centrifuged at 5000 rpm for 5 min, resuspended in PBS, and used to infect BMDM, RAW264.7, or isogenic *HDAC1* KD cells at a multiplicity of infection (MOI) of 5 or treated with 2-AA (200 μM).

### Gentamicin protection assay.

To assess bacterial clearance of P. aeruginosa in macrophages, the same clinical isolate PA14 (Rahme Lab) (55) and isogenic mutants *mvfR*, *pqsA*, or *pqsBC* were used (Rahme lab) (10, 56, 57). Bacterial strains were grown to OD_600nm_ 2.0 and used for the infection of RAW264.7 or *HDCA1* KD cell lines. Macrophage cells were plated overnight on 100-mm cell culture-treated dishes. Subsequently, cells were washed with DMEM and infected with bacteria at 5 MOI for 30 min at 37°C in 5% CO2. Unbound bacteria were removed by one wash with cold DMEM medium, and to remove any excess extracellular bacteria, 100 μg of gentamicin was added for 30 min; cells were washed with DMEM and transferred to free medium without gentamicin and kept at 37°C in 5% CO_2_. Infected RAW cells were scraped at the indicated time points (1, 2, and 3 h), centrifuged at 500 × *g*, and lysed in distilled water. The lysed cells were immediately diluted in PBS and plated on LB agar plates to assess bacterial presence. Bacterial CFU were counted after incubating the plates overnight at 37°C.

### Compounds supplementation and pharmacological inhibitors.

For experiments with 2-AA supplementation, 200 μM this compound was added. RAW264.7 cells were supplemented with 50 μM palmitoyl-CoA, stearoyl-CoA, or ATP 3 h prior to the infection. All three compounds were added after washing and immediately after infecting the cells. Rapamycin (10 μM; Sigma-Aldrich) or SCD1 inhibitor A939572 (10 μM; Calbiochem) was added to the RAW 264.7 cells 3 h prior to infection, washed, and subsequently infected.

### Cholera toxin B staining for confocal microscopy.

BMDM, RAW264.7, or isogenic HDAC1 KD cells were grown on three-chamber glass slides (IBidi cat no. 80381) overnight. Macrophages were either subjected to infection or 2-AA treatment, as described earlier. At the end of the experiment, the glass slides were placed on ice and fixed with ice-cold 4% paraformaldehyde. Immediately after, the cells were incubated with ice-cold (1 μg/mL) Alexa594-cholera toxin B subunit conjugate (Alexa594-CTB) for 30 min. Cells were washed three times with PBS, counterstained with DAPI, and examined using a confocal microscope (Nikon ECLIPSE Ti2; Nikon Instruments Inc., Tokyo, Japan) at ×400 optical magnification. The assay was conducted in triplicate and repeated at least twice.

### Immunocytochemistry.

BMDM or RAW264.7 cells were seeded at 1 × 10^5^ cells per well on three-well chambered cover glass slides the day prior to an experiment. Cells were treated with PBS (control condition) or 200 μM 2-AA or infected with PA14 or isogenic mutants *mvfR*, *pqsA*, and *pqsBC* at an MOI of 5 for 3 h as described in the previous sections. At the end of the experiment, cells were fixed in 4% paraformaldehyde in 1× PBS (pH 7.4) for 15 min at room temperature. Slides were washed in 1× PBS (pH 7.4) and incubated with blocking solution (2.5% BSA, 0.05% Triton X-100, and 1× PBS [pH 7.4]) for an additional 1 h at room temperature. LC_3_A/B (catalog no. 13082S; Cell Signaling Technology; 1:100) or SCD1 primary antibody was added to designated wells overnight at 4°C protected from light. After being washed three times with PBS-T buffer, the cells were incubated in fluorescent-labeled respective secondary antibodies. Cells were stained with DAPI (1:10,000) at room temperature and washed in 1× PBS (pH 7.4) three times for 5 min each. The cells were examined using a confocal microscope (Nikon ECLIPSE Ti2; Nikon Instruments Inc., Tokyo) at ×400 optical magnification. The assay was conducted in triplicate and repeated twice.

### Quantification of images.

Confocal images from each condition were collected using a confocal microscope (Nikon ECLIPSE Ti2; NIS-Elements 5.21; Nikon Instruments Inc., Tokyo, Japan). Fluorescence intensity from the red channel and the blue channel was measured using the ImageJ software. Fluorescence intensity arbitrary units were calculated by normalizing the red fluorescence of SCD1, LC_3_B, or CtxB values with the corresponding blue fluorescence values representing DAPI stain. Values from at least three experimental replicates were plotted using GraphPad Prism.

### RNA isolation and quantitative RT-PCR.

Total RNA was isolated from approximately 1.2 × 10^6^ cells with the RNeasy minikit (Qiagen, USA), and cDNA was prepared with the iScript Reverse transcription kit (Bio-Rad, USA), as per the manufacturer’s instruction. Real-time PCR was conducted using the PowerUP SYBR green Master mix (Applied Biosystem, USA) and primer sets for mouse *Scd1* (forward: AAGATATTCACGACCCCACC; reverse: CAGCCGTGCCTTGTAAGTTC) and mouse *Beclin1* (forward: CAGCCTCTGAAACTGGACACGA; reverse: CTCTCCTGAGTTAGCCTCTTCC) and *ULK1* (forward: GCAGCAAAGACTCCTGTGACAC; reverse: CCACTACACAGCAGGCTATCAG) and mouse 18S rRNA (forward: GTTCCGACCATAAACGATGCC; reverse: TGGTGGTGCCCTTCCGTCAAT). The transcript levels of all the genes were normalized to 18S rRNA with the Δ*C_T_* method (58). The relative expression was calculated by dividing normalized transcript levels of infected cells by those in uninfected cells. The assay was conducted in triplicate; means and standard deviations were calculated for each group.

### Western blot.

Whole-cell lysates were prepared in RIPA lysis buffer (Cell Signaling Technology, USA) supplemented with a protease inhibitor cocktail (Sigma). The concentration of the whole-cell total proteins was determined from each sample using the Bradford protein assays kit (Bio-Rad Laboratories, Hercules, CA, USA). Fifteen to twenty-five micrograms of the whole-cell lysates (total proteins) were added to 1× Laemmli buffer, boiled for 10 min, separated by SDS 7.5 to 15% PAGE in 25 mM Tris/250 mM glycine/0.1% SDS buffer (Bio-Rad), and transferred to PVDF membranes (Bio-Rad). After blocking with 5% BSA in TBS containing 0.1% Tween 20 for 1 h at room temperature, the membranes were incubated overnight with the primary antibodies specific for p62 or LC_3_B (Cell Signaling Technology), at a dilution of 1:1,000, and mouse anti-β-actin (Santa Cruz Biotechnology) at a dilution of 1:2,000. After being washed, the membranes were incubated with an anti-rabbit secondary antibody and mouse antibody for β-actin. The bands were detected by SuperSignal West Pico Chemiluminescent Substrate (Thermo Scientific) reaction, according to the manufacturer’s instructions. The gels were visualized in the ChemiDOC Imaging system (Bio-Rad Laboratories, Inc., Hercules, CA, USA). The bands were analyzed densitometrically using QuantityOne software (Bio-Rad).

### Chromatin immunoprecipitation-qPCR.

Cells were cross-linked in 1% methanol-free formaldehyde for 10 min and then placed in 0.125 M glycine for 5 min at RT. Using the truChIP High Cell Chromatin Shearing kit (Covaris, United States), cells were prepared for sonication according to the Covaris protocol. Approximately 1 × 10^7^ cells were plated in a 12 mm × 12 mm tube and subjected to shearing with the Covaris S220 sonicator for 8 min (140 peak power, 5 duty factor, 200 cycles/burst). The Magna ChIP A/G kit (Millipore, United States) was used for the subsequent immunoprecipitations according to the manufacturer’s protocol. Briefly, chromatin from approximately 10^6^ cells was incubated overnight at 4°C with 4 μg of anti-acetyl H3K18 (Abcam, USA) ChIP-grade antibody and 20 μL of A/G magnetic beads. The beads were washed serially (5 min each) with low-salt wash buffer, high-salt wash buffer, LiCl wash buffer, and TE buffer from the kit at 4°C. Chromatin was eluted with elution buffer containing Proteinase K at 62°C for 4 h, then incubated at 95°C for 10 min. DNA was isolated by column purification (QIAquick PCR purification kit). Real-time PCR was performed with the Brilliant II SYBR green super mix (Agilent, USA) and primer sets to amplify various regions of *Scd1* (forward: GGGTCATGTGTGTCCTGTGT, reverse: GTGAGCCCAACCCTATGTCC) and *Beclin1* (forward: GACGTCACTTCTGGTCCTGG, reverse: ATCGGCTCCTTTGAACCTCG) loci. Normalized values were calculated by the percent-input method relative to the IgG. The assay was conducted in triplicate; means are reported with standard deviations.

### Statistical analysis.

Whenever applicable, at least three independent experiments were performed. The error bars denote ± SD. Statistical analysis was carried out using GraphPad Prism software. One-way ANOVA followed by Tukey’s *post hoc* test was applied. Significant differences are indicated as *, *P < *0.05; **, *P < *0.01; and ***, *P < *0.001, respectively; “ns” represents no significant difference.
