# Supplementary figures and images for: Quorum-Sensing Signaling Molecule 2-Aminoacetophenone Mediates the Persistence of Pseudomonas aeruginosa in Macrophages by Interference with Autophagy through Epigenetic Regulation of Lipid Biosynthesis
Source: mBio. 2023 Apr 3;14(2):e00159-23. doi: 10.1128/mbio.00159-23 (PMC10127747; doi:10.1128/mbio.00159-23)

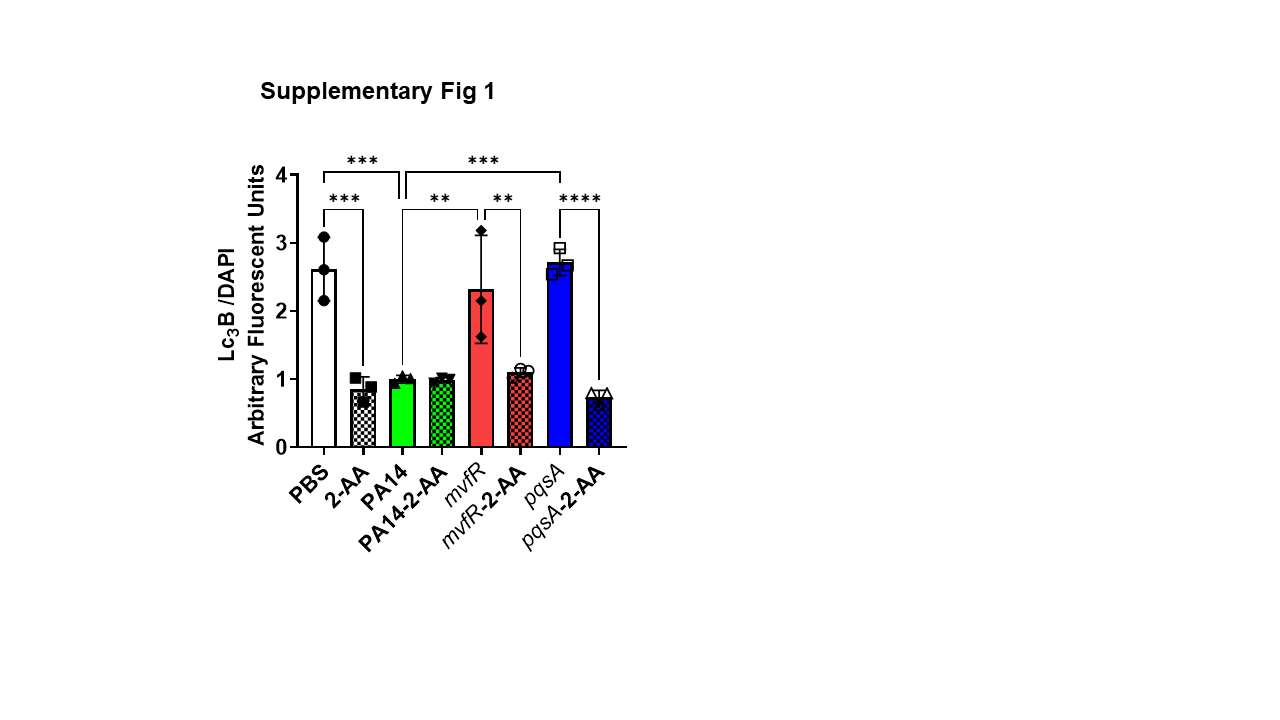

Supplement: FIG S1 [file mbio.00159-23-s0001.tif]

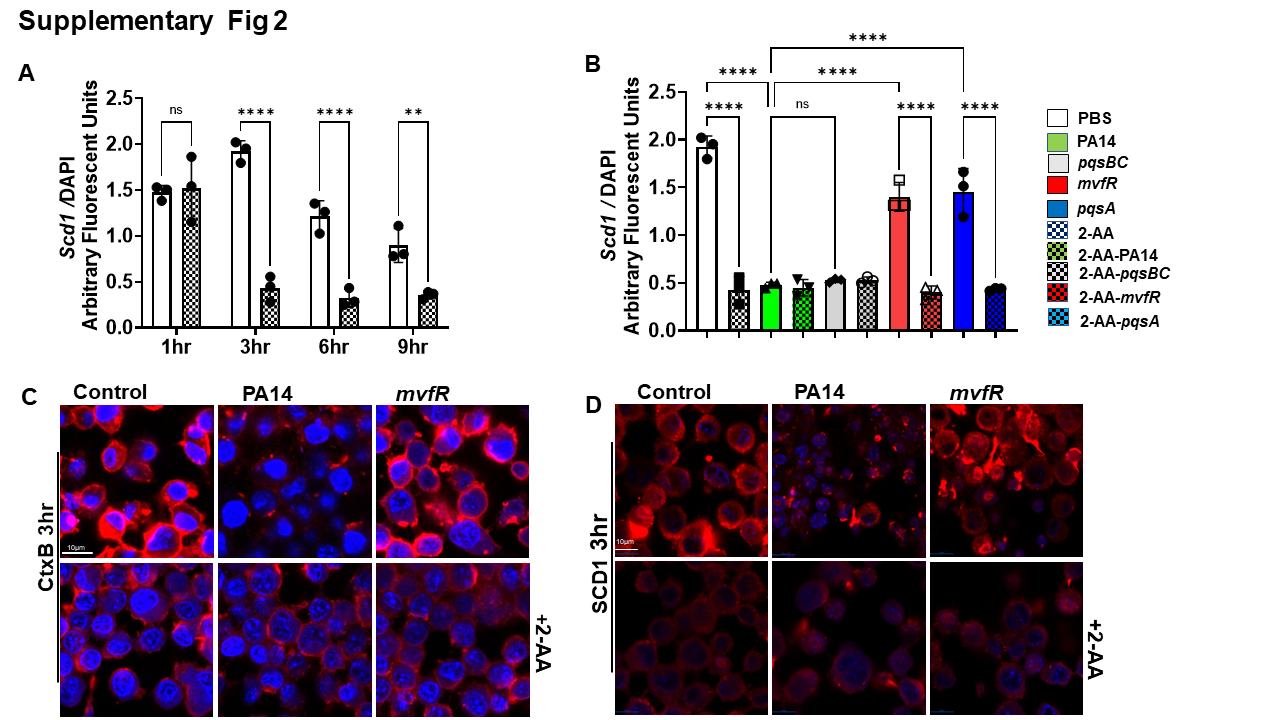

Supplement: FIG S2 [file mbio.00159-23-s0002.tif]
